# Effects of word-evoked object size on covert numerosity estimations

**DOI:** 10.3389/fpsyg.2015.00876

**Published:** 2015-07-03

**Authors:** Magda L. Dumitru, Gitte H. Joergensen

**Affiliations:** ^1^Department of Cognitive Science, Macquarie University, Sydney, NSW, Australia; ^2^University of York, York, UK; ^3^University of Connecticut, Storrs, CT, USA

**Keywords:** numerosity estimation, digit—word expression, numerical cognition, embodied cognition

## Abstract

We investigated whether the size and number of objects mentioned in digit-word expressions influenced participants’ performance in covert numerosity estimations (i.e., property probability ratings). Participants read descriptions of big or small animals standing in short, medium, and long rows (e.g., *There are 8 elephants/ants in a row*) and subsequently estimated the probability that a health statement about them was true (e.g., *All elephants/ants are healthy*). Statements about large animals scored lower than statements about small animals, confirming classical findings that humans perceive groups of large objects as being more numerous than groups of small objects (Binet, 1890) and suggesting that object size effects in covert numerosity estimations are particularly robust. Also, statements about longer rows scored lower than statements about shorter rows (cf. Sears, 1983) but no interaction between factors obtained, suggesting that quantity information is not fully retrieved in digit—word expressions or that their values are processed separately.

## Introduction

People usually count concrete objects and living things and would rather speak of “8 baskets” or of “8 elephants” than simply of “8,” for instance. Despite their frequency, these complex numerical expressions composed of a digit followed by a word referring to a concrete object have been overlooked in current research on numerical cognition. The present study is the first to investigate whether word representations impact digit values to yield combined numerosity estimations. We are particularly interested in how robust these effects are as well as in whether the two magnitudes, for digits and for words, have distinct or shared conceptual and cortical representations when processed together.

Current behavioral and neural evidence suggests that numerical abilities are flexible and depend on context, habit, and cortical development (e.g., Dehaene, 1992; Lipton and Spelke, 2003; Siegler and Opfer, 2003; Cantlon et al., 2006). Moreover, numerical abilities are common across sensory modalities (cf. Walsh, 2003) and generate interaction and interference effects with space, time, size, and luminance (e.g., Pinel et al., 2004; Cohen Kadosh et al., 2007; Conson et al., 2008). Recent research investigating the common basis of numerical values and object size (i.e., Gabay et al., 2013) has confirmed that size-congruency effects are distinct from response-initiation effects triggered by the primary motor cortex (cf. Cohen Kadosh et al., 2007) and are truly conceptual in nature. Gabay et al. (2013) used equally-sized images of small and large animals in a parity-judgment task and reported that, in conditions where response conflict effects were controlled for, images of small animals primed small numbers whereas images of large animals primed large numbers.

These findings led us to hypothesize that, when processing complex numerical expressions such as “8 elephants” or “8 ants,” the size of the objects to which the nouns refer would exert a certain influence on people’s combined numerosity estimations that is, on their concluding that both expressions refer to eight objects. So, for example, we expect that even numerate adults might unduly estimate that rows of large objects (e.g., 8 elephants) contain more members than rows of small objects (e.g., 8 ants), whereby they would be tempted to combine the two magnitude types, for digits and for objects. Indeed, numerosity estimations of concrete objects vary with object size such that groups of small objects are judged to be less numerous than groups of large objects (cf. Binet, 1890). We therefore anticipate that merely mentioning a group of objects would evoke their combined size, which in turn would affect the overall numerosity estimations of digit-word expressions.

Language instantly evokes object properties including object size (Rubinsten and Henik, 2002; Setti et al., 2009; Sellaro et al., 2015), as predicted by theories of embodied and grounded cognition (Barsalou, 2008). These theories hold that people evoke multimodal perceptual simulations during online language processing based on their experience with concrete situations. Therefore, since language expressions are grounded in situations where people routinely use them, merely reading about an object is likely to evoke a full array of related experiences that gives instant access to associated perceptual and cognitive processes. Furthermore, results from brain imaging studies indicate that the same regions become active when objects are presented in pictorial form or when they are mentioned by language (e.g., Chao et al., 1999; Just et al., 2010). Research has also found that the retrieval of number magnitude is a spontaneous process similar to automatic language processing (Paivio, 1971; Barsalou, 2008) such that numbers are rapidly assigned approximate representations prior to further refinement in specific cortical areas (e.g., Tzelgov et al., 1992).

Among the studies devoted to investigating language-evoked object size, we recall the evidence reported in Rubinsten and Henik (2002), who used a Stroop-like paradigm to show that, in physical-comparison tasks (i.e., estimating which font size is larger) as well as in conceptual-comparison tasks (i.e., estimating which real-life animal is larger), judgments were faster for congruent animal names (e.g., “lion” written in large font or “ant” written in small font) than for incongruent names (e.g., “lion” written in small font or “ant” written in large font). Similar evidence was provided by Setti et al. (2009) who used an indirect task (i.e., category decision) asking participants to decide whether two objects evoked by a prime word and by a target word belonged to the same category. People responded faster to targets following same-size primes (e.g., “elephant” following “giraffe”) than to targets following different-size primes (e.g., “hare” following “giraffe”).

In our study, we used an indirect task (i.e., property probability ratings) to explore the hypothesis that object size affects numerosity estimations in digit-word expressions. We relied on a well-established finding that people tend to evaluate single entities more positively than groups (i.e., the “person-positivity bias hypothesis” cf. Sears, 1983), which results in lower probability ratings for a particular property as groups grow larger. For example, when participants are presented with the information “There are 8 elephants in a row” or “There are 156 elephants in a row” and subsequently rate the probability that the statement “All elephants are healthy” is true, their scores should be lower for the statement about 156 elephants than for the about 8 elephants. We further predict that participants will rate small animals’ health higher than large animals’ health (e.g., “All ants are healthy” following “There are 8 ants in a row” would score higher than “All elephants are healthy” following “There are 8 elephants in a row”). In other words, adults might consider rows composed of large animals as being more numerous than rows composed of the same number of small animals and thus think of animals in “long” rows as being less healthy than animals in “short” rows. Object size effects may occur despite people’s ability to instantly recover the representation of the digit “8” in “8 ants” and “8 elephants,” for instance, because they are also able to rapidly evoke the size of the animals mentioned.

Our covert task (i.e., object-property probability ratings) taps into the later stages of combined magnitude processing hence obtaining significant effects of word-evoked object size on numerosity estimations would indicate that object-size effects are particularly robust. To further preclude confounds relating to whether size affects digit magnitude in virtue of the form of the statement rather than in virtue of the way that sentence fragments combine (i.e., jointly or independently), we varied the quantifier type to suggest aggregate (i.e., “All elephants are healthy”) as well as discrete numerosities (i.e., “Each elephant is healthy”).

## Materials and Methods

### Subjects

Fifty-two native English speakers volunteered for an online study in return for course credit.

### Stimuli

Stimuli were 36 sentences of which half included small animals (e.g., bats, mice, crabs) and half included large animals (e.g., tigers, bears, wolves), as determined from a previous rating study summarized in Table 1. Average ratings were calculated based on individual size ratings (*N* = 22) of 100 items from two categories (animals and vegetables). Participants rated the size of each item presented individually in a scale from “0” (“not very big”) to “10” (“very big”). We then selected 36 items (i.e., names of small and large animals) from the rating study such that large animals received ratings at least twice as high as small animals and were also matched for frequency and length. Each sentence was followed by a statement about the health of the animals mentioned, as explained below. We constructed two lists (Latin square design) such that all participants saw each number once, paired with a small animal in the first list and with a large animal in the second list. In each list, half of the animals were small and the other half were large. Numbers ran from 3 to 8 in short rows, from 43 and 95 in medium rows, and from 1269 to 8421 in long rows. Both the numerosity study and the preliminary rating study were conducted in accordance with the ethics requirements of the University of York and followed relevant regulatory standards.

**TABLE 1:** **Animal names in all stimuli statements used in the experimental study^a^**.

	**Big Animals**				**Small Animals**		
	***Length***	***Frequency***	***Size***		***Length***	***Frequency***	***Size***
Bears	5	3.17	7.77	Bats	4	2.74	2.95
Bisons	6	1.04	7	Bees	4	2.39	0.91
Camels	6	2.14	7.45	Beetles	7	1.76	1.14
Chimpanzees	11	1.6	5.86	Crabs	5	2.27	2.95
Crocodiles	10	1.85	6.59	Crickets	8	1.97	1.14
Deer	4	2.39	6.45	Doves	5	2.12	3
Donkeys	7	2.21	6.36	Finches	7	1.8	2.45
Foxes	5	2.61	4.59	Flies	5	3.32	0.55
Giraffes	8	1.76	8.32	Goldfish	8	2	1.77
Goats	5	2.53	4.82	Hamsters	8	1.75	2.32
Gorillas	8	2.19	7.05	Magpies	7	1.11	2.77
Hippos	6	1.6	7.77	Mice	4	2.6	2.68
Horses	6	3.19	7.05	Pigeons	7	2.26	2.95
Panthers	8	1.67	6.68	Rats	4	2.97	2.73
Reindeer	8	1.93	6.77	Robins	6	2.44	2.45
Tigers	6	2.64	7.09	Sparrows	8	1.77	2.36
Wolves	6	2.57	5.82	Spiders	6	2.36	1.45
Zebras	6	1.77	6.45	Squirrels	9	2.22	3.23
***Average Scores***	**6.72**	**2.16**	**6.66**	***Average Scores***	**6.22**	**2.21**	**2.21**

^a^20 participants rated the size of 36 animals on a scale from 1 (“not very big”) to 10 (“very big”).

Names of big and small animals were matched in length and frequency.

### Design and Procedure

**FIGURE 1:**
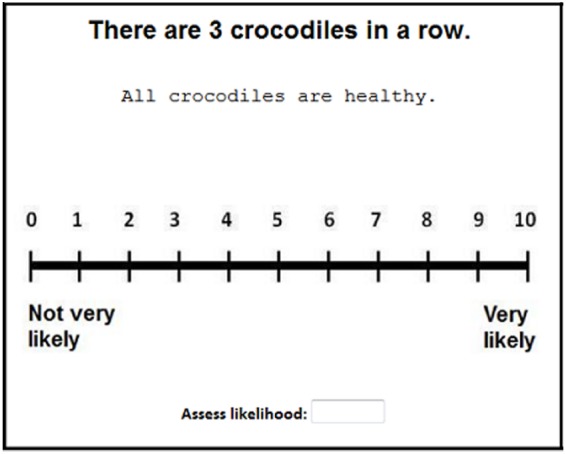
**Example stimulus in our study.** In each trial, participants read a description (e.g., *There are 3 crocodiles in a row*) followed by a statement (e.g., *All crocodiles are healthy*), whose likelihood they rated on a scale from 0 (not very likely) to 10 (very likely). In half of the trials, the statement contained a different quantifier (e.g., *Each crocodile is healthy*).

The experiment followed a 2 (Size: Small vs. Large animals) × 3 (Row-length: Short vs. Medium vs. Long) fully factorial design. We also introduced “quantifier” as a between-subjects factor such that half of the participants read statements containing the quantifier *all* and the other half read statements containing the quantifier *each*. On a typical trial, participants read a description (e.g., *There are 3 crocodiles in a row*) followed by a statement (e.g., *All crocodiles are healthy* or *Each crocodile is healthy*), which they rated on a scale from 0 (“not very likely”) to 10 (“very likely”), as seen in Figure 1.

## Results

**FIGURE 2:**
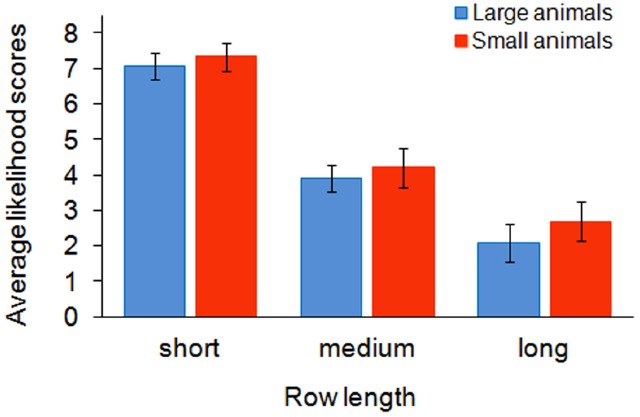
**Mean likelihood judgments and 95% CIs for statements (e.g., *All elephants are healthy*) following descriptions of short, medium, and long rows of animals (e.g., *There are 8/ 156/ 2600 elephants in a row*).** Rows of small animals were perceived as less numerous than rows of large animals, hence higher scores obtained for the former than for the latter.

Figure 2 summarizes the average likelihood scores across conditions. A 2 (Size: Small vs. Large animal) × 3 (Row-length: Short vs. Medium vs. Long) *ANOVA* revealed a main effect of size, *F*(1, 50) = 6.62, *p* = 0.013, ${\text{η}}_{p}^{2}$ = 0.117, and a main effect of row-length, *F*(2, 100) = 173.76, *p* < 0.001, ${\text{η}}_{p}^{2}$ = 0.777, but no interaction between factors, *F*(2, 100) = 2.06, *p* = 0.132, suggesting that group size as well as word-evoked object size influence property-probability ratings and thereby covert numerosity estimations.

We also calculated Cohen’s d for each row-length condition separately and found a sizeable difference between the effect size in the long-row condition and the effect size in the short- and medium-row conditions, namely a value of 0.311 for long rows, a value of 0.183 for short rows, a value of 0.123 for medium rows, suggesting the existence of a qualitative distinction between small and medium groups comprising at most tens of individuals on the one hand, and very large groups comprising thousands of individuals on the other hand.

Importantly, we found no effect of quantifier type, *F*(1, 50) = 0.189, *p* = 0.665, suggesting that magnitude estimations were not dependent on whether the quantifiers accompanying animal names prompted participants to view the groups (i.e., rows of animals) as aggregates (i.e., the quantifier “all”) or as discrete sums of individuals (i.e., the quantifier “each”).

## Discussion

We provided evidence that word-evoked object size impacts numerosity estimations in a covert task where participants rated the probability that several objects (i.e., 8 elephants) mentioned in a previous statement are healthy. We obtained a main effect of object size such that participants rated health statements about large animals lower than health statements about small animals, thereby confirming previous findings that language evokes object size, which in turn impacts number processing (Rubinsten and Henik, 2002; Setti et al., 2009; Gabay et al., 2013; Sellaro et al., 2015). Unsurprisingly (cf. Sears, 1983), we also obtained a main effect of group size such that health statements about long rows of animals scored lower than statements about medium rows, which in turn scored lower than statements about short rows.

Interestingly, we observed no interaction between factors, which might suggest that quantity information is not fully retrieved in digit—word combinations or that digit and word magnitudes are processed separately at some level. Indeed, current evidence suggests that de-composition may occur for expressions containing same-type magnitude values, in particular for two-digit combinations (e.g., Nuerk et al., 2001) such that each digit is processed separately. Unfortunately, a decomposition account of same or different magnitude types runs counter previous evidence (i.e., size congruency effects in reaction-time studies) supporting a shared magnitude code across quantity dimensions. Nevertheless, the predictions of the decomposition account and of the size-congruency principle could be reconciled if we examined more closely the particularities of our task and associated cognitive processes.

Most notably, the effect of object size is robust but small that is, numerical estimations of digit—word expressions are largely determined by digit values, which are subsequently modulated by the size of a single object rather than by the combined size of a group whose cardinality matches the digit value. In other words, the plural form on the noun in “8 ants” does nothing to influence overall numerosity estimations, which suggests that language processing constraints might be responsible for the lack of interaction between object number and object size. In particular, linearity requires that items in a string be processed one by one in the order in which they are mentioned and is thus compatible with the so-called “anchoring bias” (cf. Tversky and Kahneman, 1974), which is a general-cognitive tendency toward grounding upcoming information into information already acquired. In digit-word expressions, the information provided by digit representations serves to anchor subsequent information provided by word representations, with lasting effects. In particular, our covert numerosity task (i.e., property probability ratings) explored the late combination stages of word-evoked object size and overall numerosity estimations rather than early behavioral reactions in item-by-item processing, as was the case in previous studies. The linearity constraint is likely to be responsible for the incomplete retrieval of quantity information. It is a matter for further research to confirm this hypothesis as well as whether full magnitude retrieval might be obtained for languages with a different word order, namely for languages where digits follow object names.

Let us now briefly consider the score differences between the short and medium row conditions on the one hand and the large row condition on the other hand, which were rather sizeable in the absence of a significant interaction between object number and object size. We believe that these findings too are amenable to task properties, in particular to the stimuli used (i.e., digit magnitudes). Unlike previous studies where small numbers ran from 1 to 10 and large numbers would not surpass 100, our study included extremely large values (i.e., thousands) in the long-row category, which people might find less familiar or more difficult to grasp. The qualitative properties of very large magnitudes are likely to result from the comprehension effort they require, which might help explain why score differences between small and large animals were greatest in effortful trials (i.e., in the “long row” condition). By comparison, the tasks used in previous behavioral and neuro–cognitive studies reporting significant object size effects strongly evoked motor control and were thus inherently effortful. Importantly, effortful processing depends on participants’ goals hence specific cortical areas are recruited for handling the response types required. These findings suggest that the mapping between number magnitude and action representation is rather flexible (Koch and Prinz, 2005; Koch and Rumiati, 2006; Wenke and Frensch, 2005). Indeed, as shown in Fias et al. (2001) and in Lammertyn et al. (2002), effects of Spatial-Numerical Association Response Code (SNARC – e.g., Dehaene et al., 1993) were obtained only when participants judged the orientation of a digit, but not when they judged the color of the digit, arguably because the processing of numbers as well as orientation relies on regions of the parietal cortex, which belongs to the dorsal stream, while color processing relies mainly on regions of the inferior temporal cortex, which belongs to the ventral stream (Zeki et al., 1999). Since particular tasks involve different magnitude representations in the ventral and dorsal pathways, the extent of their neural overlap determines the interaction between numbers and action as well as between numbers and space (e.g., Badets et al., 2007).

In the present study, the object size effect as well as the qualitative difference between small and medium groups on the one hand and large groups on the other hand might stem from a basic tendency toward translating different magnitude types onto each other as well as from an instant appraisal of the effort required for manipulating the objects, as predicted by theories of embodied cognition (Barsalou, 2008), thus engaging specific cortical pathways. It remains an issue for future research to carefully determine the relevance of the manipulability hypothesis (e.g., Moretto and Di Pellegrino, 2008; Badets and Pesenti, 2010, 2011; Ranzini et al., 2011) for the processing of digit-word expressions by varying response type and/or object affordability (e.g., manipulable vs. non-manipulable).

Numerate adults’ susceptibility to object-size biases also remains to be investigated in future research. Whereas it is widely acknowledged that the number sense is influenced by maturation levels, which generate differences in cortical activity between children and adults (Dehaene et al., 2003; Cantlon et al., 2006; Hyde et al., 2010), the extent to which maturation levels reflect expertise levels is largely unknown. The existence of correlations between maturation and expertise levels might help explain why children’s ability to discriminate numerosities and their capacity to map numbers onto distinct numerosities are not perfected before adolescence, once they have been exposed to a full range of numerical information (e.g., Lipton and Spelke, 2003). We believe that, in our study, adults’ numeracy expertise has prevented them from unduly concluding that the result of counting 8 elephants would be very different from the result of counting 8 ants, thus yielding only small effects of object size and no interaction between number and size. In other words, though object size exerted only a limited influence on adults’ numerosity estimations, it might have a greater impact on children and adults who lack extensive expertise with numerical calculations (e.g., tribal populations). The results of our study suggest that words can readily evoke object properties, which numerate adults factor in when making overt property likelihood judgments and thereby covert numerosity estimations.

### Conflict of Interest Statement

The authors declare that the research was conducted in the absence of any commercial or financial relationships that could be construed as a potential conflict of interest.
